# DNA Nanotechnology on Bio-Membranes

**DOI:** 10.3390/membranes12050491

**Published:** 2022-04-30

**Authors:** Bin Zhao, Mingxu You

**Affiliations:** 1 Department of Chemistry, University of Massachusetts, Amherst, MA 01003, USA; 2 Carl R. Woese Institute for Genomic Biology, University of Illinois at Urbana-Champaign, Urbana, IL 61801, USA; 3 Department of Biochemistry and Molecular Biology, University of British Columbia, Vancouver, BC V6T 1Z3, Canada; 4 Molecular and Cellular Biology Program, University of Massachusetts, Amherst, MA 01003, USA

In recent years, DNA nanotechnology, including both structural and dynamic DNA nanotechnology, has emerged as a powerful tool for various analytical and biomedical applications in biological membranes. Based on the programmable and predictable self-assembly and innate biocompatibility of DNA molecules, structural DNA nanotechnology has built numerous 2D and 3D nanostructures with unprecedented precision and complexity. On the other hand, dynamic DNA nanotechnology focuses on the creation of nanoscale devices with designed dynamic functionalities to undergo controlled motion or reconfiguration. These DNA nanostructures and nanodevices have started to be extensively exploited to engineer biological membranes for a variety of applications, including analytical and biophysical characterization, the regulation of membrane signaling and interactions, and transmembrane deliveries of nucleic acids.

This Special Issue titled “DNA Nanotechnology on Bio-Membranes” aims to cover recent developments and advances in all aspects of applying DNA nanotechnology to biological membranes. Different topics have been discussed, including the design and fabrication of membrane-interacting DNA nanostructures, the study of stability of lipid-based polymer amphiphiles, an overview of DNA-based molecular engineering of cell membrane, and the application of DNA nanotechnology in obtaining precise molecular information. There are four contributions, namely two research articles and two reviews, in this Special Issue.

Singh et al. [1] optimized design parameters to minimize cholesterol-induced aggregation of membrane-interacting DNA origami nanostructures. Cholesterol-modified DNA origami has been widely used for bio-membrane engineering and modification. Nevertheless, the parameters that can reduce unwanted cholesterol-induced aggregation of DNA origami nanostructures have not been systematically studied. To fill this gap, the authors evaluated the folding yield of monomeric 2D cholesterol-DNA origami tile (DOT) and 3D cholesterol-DNA origami barrel (DOB) based on different design parameters. It was found that the DOT assembly yield could be significantly increased by decreasing the spacer length connecting cholesterol and DOT (from 38% to 95%) or reducing the number of cholesterols from 6 to 1 (2% to 100%). Their results also indicated that the use of protective 10T overhangs or separation between cholesterol groups could result in an increase in the DOT-folding yield. Similarly, the DOB assembly yield could also be improved by reducing the number of cholesterols. This work has provided some important general guidelines in reducing the cholesterol-induced aggregation of membrane-interacting 2D and 3D DNA origami nanostructures.

In another study, Yu et al. [2] reported the effect of molecular structures on the cell surface stability of lipid–polymer amphiphiles under different mechanical stresses. Lipid–polymer amphiphiles are typically composed of a hydrophobic lipid anchor, a solubility promoting polymer, and a functional component (e.g., aptamers). These lipid–polymer conjugates are capable of exogenously and spontaneously inserting onto the plasma membranes without affecting cellular functions and viability. These lipid–polymers have been known to stably tether on the cell surfaces under static conditions. However, their stability under mechanical shear stress (e.g., in the blood flow) remains largely uninvestigated. In this study, the authors carefully analyzed the effect of different molecular structures of diacyl lipid-polyethylene glycol amphiphiles on their kinetic stability on the surface of red blood cells under mechanical shear stress. It was found that long polyethylene glycol could be released at a higher rate than short polymers under the same shear stress both in vitro and in vivo. Meanwhile, cationic lipid facilitates the long-term retention of amphiphilic cargos on the membranes of red blood cells during circulation.

In a review article, Li et al. [3] summarized some recent advances in applying nucleic acids for cell membrane engineering, particularly focusing on molecular sensing and imaging, cell–cell communication and activity regulation, and biomimetic membrane structures. Functional nucleic acids, such as aptamers and DNAzymes, have proven to be powerful for cell surface sensing and imaging due to their flexible design and ease of modification. By conjugating with hydrophobic lipids, these functional nucleic acids have been used for monitoring extracellular pH, metal ions, neurotransmitters, and signaling molecules in cellular microenvironments. Of note, lipid-modified DNA probes can also be anchored on the live cell membrane for monitoring dynamic molecular encounters and sensing intercellular mechanical forces. Another major application of membrane anchored-DNA probes was to mimic and manipulate cell–cell interactions for immunotherapy or stimuli-responsive regulation. DNA-based molecular probes have also shown great potential for monitoring and regulating cell surface receptors. Furthermore, DNA nanostructures are ideal materials for constructing biomimetic membranes and nanopores for controlled target transportation. 

Lastly, Tang et al. [4] reviewed recent progress in using DNA nanotechnology to obtain precise molecular information, particularly in the structural determination and reconstruction of proteins, protein–protein interactions, and measurement of molecular forces. For example, a self-assembled DNA nanobarrel was designed as an in vitro lipid scaffold to reconstitute a single membrane protein: alpha-hemolysin. Furthermore, to reconstruct larger sized membrane proteins for structural determination, lipid-decorated DNA-corralled nanodisc can also be devised. As another example to map the nano-environment and interactions among membrane proteins, a non-microscopy-based method for the in situ ensemble analysis of membrane protein nanodomains (such as Her2) was developed based on a DNA nanocomb structure on SK-BR-3 and MCF-7 cell membranes. In addition, cell surface protein receptors can mediate and transmit mechanical forces between the cell and its microenvironment. DNA-based tension probes have been rationally designed to measure these piconewton-level forces. Moreover, dynamic mechanical information storage could be achieved using these DNA probes to enable the imaging of short-lived cellular forces.

In conclusion, the exciting findings and critical discussions from these contributions highlighted the unique and critical roles of DNA nanotechnology on biological membranes. Numerous DNA nanostructures and nanodevices demonstrated their excellent capabilities in membrane applications. This Special Issue provides general guidelines and in-depth information to facilitate further developments of this emerging field of DNA nanotechnology on bio-membranes.
